# Development of ARDS after Excessive Kath Consumption: A Case Report

**DOI:** 10.1155/2011/291934

**Published:** 2011-07-24

**Authors:** Marlene Wewalka, Andreas Drolz, Katharina Staufer, Thomas M. Scherzer, Valentin Fuhrmann, Christian Zauner

**Affiliations:** ^1^Intensive Care Unit, Department of Gastroenterology and Hepatology, Medical University of Vienna, Waehringer Guertel 18-20, 1090 Vienna, Austria; ^2^Intensive Care Unit 13H1, Department of Internal Medicine III, Medical University of Vienna, Waehringer Guertel 18-20, 1090 Vienna, Austria

## Abstract

Khat is a drug widely used in the Horn of Africa and the Arabian Peninsula. Khat leaves contain, among other substances, the psychoactive alkaloid cathinone, which induce central nervous system stimulation leading to euphoria, hyperactivity, restlessness, and insomnia. However, it also could cause psychological adverse effects such as lethargy, sleepiness, psychoses, and depression necessitating pharmacologic treatment. Here we report the case of a 35-year-old man from Somalia who became unconscious and developed aspiration pneumonia and subsequent ARDS after excessive consumption of khat leaves. His unconsciousness was possibly caused by the sleepiness developed after khat consumption and a benzodiazepine intake by the patient himself. Thus, khat-induced adverse effects should not primarily be treated pharmacologically, but patients should be urged to quit khat consumption in order to eliminate or, at least, reduce the severity of present psychological adverse effects.

## 1. Introduction

Khat (*Catha edulis *Frosk.) is a scrub native in the Horn of Africa and the Arabian Peninsula. In these areas chewing its fresh leaves is widespread as a social habit, leading to euphoria, alertness via central nervous system stimulation [1]. Therefore, khat was used traditionally to relieve fatigue, and it had occupied some place in self-medication of obesity and depression [1, 2]. As a result of migration khat has gained global availability [1]. For the present, the prevalence of khat chewing in Western countries appears to be restricted to the immigrant communities from these countries [3]. However, there is some evidence that khat use has spread to the host population also, and a pill containing an extract of khat leaves, known as “Hagigat,” has been sold to Israeli drug users [4, 5]. 

The mentioned central stimulant actions are evoked primarily by cathinone, the main active alkaloid in fresh picked leaves, and it is structurally similar to D-amphetamine [1]. Cathinone is considered as an indirectly acting sympathomimetic alkaloid having catecholamine-releasing properties at both central serotoninergic and dopaminergic synapses as well as at peripheral noradrenaline storage sites [6]. It is structurally similar to amphetamine and operates through the same mechanism [7]. Thus, cathinone induces pronounced behavioral effects including euphoria, excitability, anxiety, irritability, hyperactivity, restlessness, and insomnia in a dose-related manner [8, 9]. This is followed by lethargy and sleepiness in the next morning. Khat usage is also associated with memory impairment, depression, psychoses, and psychological dependence [9, 10]. However, khat chewing not only affects the central nervous system, but also impairs other organ systems like the cardiovascular and the cerebrovascular system, the gastrointestinal tract, and the liver as well as other peripheral organ systems [1, 6]. Thus, khat chewing is able to cause substantial health damage, and it could have social, financial as well as medical consequences [3, 11]. Here we present a case report of a 35-year-old male who developed aspiration pneumonia with subsequent ARDS after excessive consumption of khat leaves.

## 2. Case Presentation

A 35-year-old male patient was admitted to our medical intensive care unit (ICU) because of multiple organ failure. 

The patient was born in Somalia and migrated to Austria six years ago. In his past history there were tuberculosis detectable about 15 years ago, which was treated successfully, and unspecific heart problems. Because of insomnia and depression, a tricyclic antidepressant (amitriptyline) and diazepam were prescribed. In addition, every day the patient chewed khat leaves. According to his next of kin, khat intake increased considerably during the past few days before hospital admission. In this context, they reported that the patient was sleeping nearly until noon. However, on the day of hospital admission it was difficult to rouse the patient even in the early afternoon. Hence, an emergency physician was called to the patient, who found him neurologically disturbed with the risk of aspiration. Thus, the patient was sedated, intubated, and transferred to the emergency department (ED) of our hospital thereafter. A CT scan of the brain was performed to exclude any underlying structural cause within the brain for the present unconsciousness. At the ED an electrocardiogram showed arterial fibrillation with a heart rate of 143 bpm. Conversion into sinus tachycardia was achieved spontaneously within six hours subsequently. Hemodynamics was stable during that time period. However, gas exchange was considerably compromised. After the performance of bronchoscopy, which showed no foreign bodies or massive secretions but reddened mucosa indicating aspiration of gastric acid, the patients was transferred to our medical ICU. Further clinical and laboratory data at ED admission as well as at ICU admission are presented in Table 1.

At ICU admission SOFA score was found to be 8, and SAPS II score was 46. Hemodynamic condition was stable without catecholamine support. Moreover, during ICU stay the patient developed arterial hypertension, which was treated accordingly. The patient remained intubated and under analgosedation (combined propofol and remifentanil administration) administered continuously. Pulmonary function remained significantly compromised due to present aspiration pneumonia, which proceeded into ARDS over the next few days. Therefore, transient prone positioning of the patient was performed. However, gas exchange improved under antibiotic therapy that the patient could be extubated after two weeks of mechanical ventilation. 

After the cessation of the mechanical ventilation the patient showed massive agitation indicating present delirium, which could be improved by the administration of clonidine as well as lorazepam and trazodone. Finally, the patient was discharged from the ICU after 22 days and discharged from the hospital in good condition another 14 days thereafter.

## 3. Discussion

Khat chewing is widely practiced in the Horn of Africa and the Arabian Peninsula. Nonetheless, due to easy transportation and easing of importation restrictions khat is spreading to Western countries in recent years [1, 3]. Khat leaves contain cathinone, a psychoactive alkaloid, which is structurally similar to amphetamine [1]. Thus, it induces pronounced behavioral effects by stimulation of the central nervous system [8, 9]. However, it provokes systemic adverse effects on different organ systems such as psychological or cardiovascular as well [1, 9]. Thus, cathinone could entail substantial damage of health accompanied by grave social and economical consequences [1, 3, 6, 10]. 

Our patient presented atrial fibrillation with a heart rate up to 143 bpm. Tachycardia and cardial arrhythmias are well known adverse effects seen in khat users [9]. These adverse effects are possibly caused by stimulation of trace amine-associated receptors by cathinone [1, 12]. 

Cathinone, a psychoactive alkaloid found in khat leaves, also has a stimulating effect on the central nervous system inducing psychological sensations such as euphoria, hyperactivity, restlessness, and insomnia [8, 9]. However, these symptoms are followed by lethargy and sleepiness. Because of these side effects, which can be observed in the morning after khat consumption, the family of the patients was not concerned that he was sleeping during the whole morning. Since it was difficult to rouse the patient even in the early afternoon an emergency physician was called, who found the patient comatose with insufficient adverse effects reflexes. Thus, the patient was intubated and transferred to the ED, where an underlying cause located in the central nervous system was excluded by a CT scan of the brain. A chest X-ray, however, showed an infiltration in the left-sided lower lobe of the lungs conformable with aspiration pneumonia. This pneumonia progressed to ARDS over the next few days necessitating prolonged artificial ventilation accompanied with intermittent prone positioning of the patient. 

Nonetheless, there are no reports that khat could induce coma in chronic khat consumers, which would explain aspiration seen in our patient as a single cause. Thus, it is likely that the prescribed medication of the patient was also responsible for that disturbance to a certain extent. Due to pre existing depression as well as insomnia amitriptyline and diazepam have been prescribed several times before the accident. Amitriptyline was taken regularly, which was confirmed at admission by a plasma concentration within the normal range. Nonetheless, the patient used additional diazepam on demand in order to calm himself after excessive khat use. Thus, it is possible that the combination of sleepiness after khat consumption and an intake of diazepam effectuated coma, which caused aspiration to the lungs subsequently. 

Insomnia, psychoses as well as depressive mood are known adverse effects of khat consumption [8–10]. Thus, one should prescribe hypnotic drugs very carefully to treat these adverse effects, because such drugs could potentiate lethargy and sleepiness, which could lead to unconsciousness. Patients should be urged to quit khat consumption in order to eliminate or, at least, reduce the severity of the mentioned psychological disorders and khat-induced adverse effects should not primarly be treated pharmacologically. 

## Figures and Tables

**Table 1:** Clinical and laboratory data on admission to the ED as well as to the ICU.

	Normal range	ED admission	ICU admission
HR, bpm	—	143	117
MAP, mmHg	—	65	77
PaO2/FiO2	—	72,7	120
Body temperature, °C	—	38.1	37.9
Lactate, mmol/L	0.5–1.6	3.4	1.4
Creatinine, mg/dl	<1.2	1.95	1.41
GOT, U/L	<35	202	290
GPT, U/L	<45	248	320
gGT, U/L	<55	316	238
CK, U/L	<190	1014	1553
Troponin T, ng/ml	0–0.03	0.274	Na
CRP, mg/dl	<1	5.51	9.27
WBC, G/L	4.0–10.0	10.35	8.77
